# Severe Oral Mucosal Ulceration Associated with Oral Bisphosphonate Use: The Importance of Imparting Proper Instructions on Medication Administration and Intake

**DOI:** 10.1155/2021/6620489

**Published:** 2021-03-10

**Authors:** Manju Chandran, Wanling Zeng

**Affiliations:** ^1^Complicated Metabolic Bone Disorders Clinic, Osteoporosis and Bone Metabolism Unit, Department of Endocrinology, Singapore General Hospital, Bukit Merah, Singapore; ^2^Department of Endocrinology, Singapore General Hospital, Bukit Merah, Singapore

## Abstract

Oral bisphosphonates are approved for the treatment of bone loss associated with several conditions including postmenopausal osteoporosis. Although generally well tolerated, adverse effects such as gastroesophageal reflux and oesophageal and peptic ulceration may occur. Oral mucositis and ulceration are lesser-known side effects. Proper counselling and rigorous adherence to the administration instructions are crucial. We describe a case of bisphosphonate-induced severe oral mucosal ulceration in an elderly woman that was caused by incorrect instructions and/or incorrect understanding of instructions for oral alendronate intake.

## 1. Introduction

Oral bisphosphonates such as alendronate and risedronate are amongst the most commonly prescribed medications for the treatment of osteoporosis in men over the age of 50 and in postmenopausal women. They have been in clinical use for this purpose since 1970 [1]. Dental and orthopaedic surgeons as well as physicians involved in the care of osteoporosis are well aware of bisphosphonate-related osteonecrosis of the jaw (BRONJ), a rare but calamitous problem that has been associated with oral and intravenous bisphosphonates [2, 3]. Patients are also usually counselled on gastric and oesophageal side effects of the oral bisphosphonates and are instructed to stay upright for at least 30–45 minutes after taking these oral agents to avoid esophagatis. Less well-known side effects are the oral mucosal lesions that may be associated with the use of these agents of which very few reports in the scientific literature databases exist. Most of the ones reported were in situations where the patients could not follow the instructions given for proper intake of the medication due to associated mental and or physical comorbidities [4–6]. We describe a case of severe oral mucosal ulceration in a patient with no such comorbidities which is presumed secondary to inadequate instructions about proper intake of the medication. The mucositis had a clear temporal association with intake of oral alendronate tablets. Written permission was obtained from the patient for taking anonymized photographs of her. Permission was also obtained to write and submit a case report to a scientific journal.

## 2. Case Report

**Figure 1:**
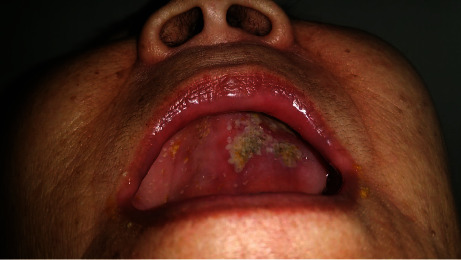
Appearance of the oral cavity on the 19^th^ of September prior to the second dose of alendronate. Mucosal ulceration with some sloughing noted on the hard palate.

**Figure 2:**
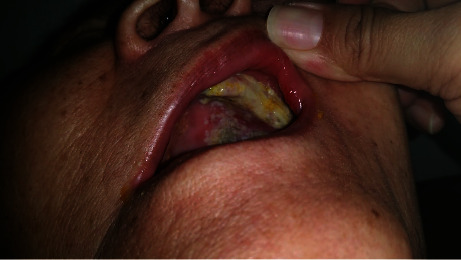
Appearance of the oral cavity on the 21^st^ of September with extension of oral ulcer to the upper gum and sloughing.

A 79-year-old Chinese female accompanied by her daughter was seen at the complicated metabolic bone disorders clinic of our hospital for a second opinion on the management of her severe osteoporosis. She stated that she had been started on oral alendronate about two weeks ago, but had developed an “allergy” to it and therefore wanted an alternative medication. She had a history of left ankle fracture in 2017. Axial dual energy X-ray absorptiometry (DXA) scan of the hip and spine showed a T score of −3.8 at the left femoral neck and −3.5 at the lumbar spine. She had no other medical problems other than dyslipidemia for which she had been taking lovastatin for several years. She had been started on generic alendronate tablet 70 mg/week by the physician. She took the first dose on 12 September. On 13 September, she started to experience gum pain with food intake and noticed a small oral ulcer of approximately 1 cm on the hard palate. She dismissed it without giving it much importance. By the 19^th^ of September, when she took the 2^nd^ dose of alendronate, the ulcer had increased in size (Figure 1). By now, the oral pain had increased, and she found it difficult to take any food that was not soft or sweet. The ulcer had increased in size and had extended to the upper gum (Figure 2). She went back to the general practitioner on 21 September who diagnosed her with oral thrush and prescribed an antifungal oral gel. The ulceration by then had progressed rapidly to involve the tongue and the inside of the left side of the lip, and she could only tolerate a liquid diet. She had a fever with temperatures up to 38.5 degree Celsius that lasted for about 8 hours that subsided with oral acetaminophen tablets. She had no previous history of oral ulcers or gastric problems. No skin lesions were present. Other mucosal surfaces such as the eyes and genitalia were not affected.

**Figure 3:**
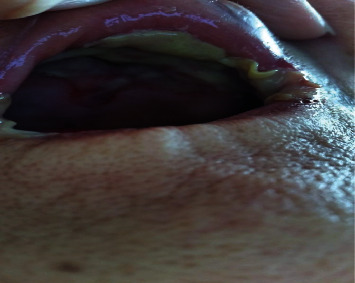
Appearance of oral cavity on the 22^nd^ of September (10 days after the first dose of alendronate) with extensive involvement of the oral mucosa and angles of the mouth.

**Figure 4:**
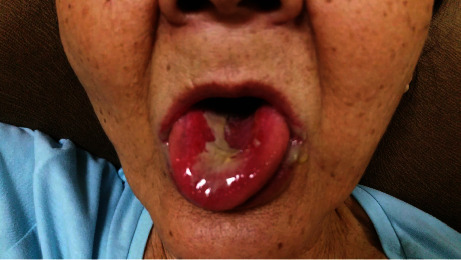
Appearance of the oral ulcer on the 25^th^ of September upon consultation at the complicated metabolic bone disorders clinic. 3 × 4 cm sloughy erosion with irregular margin on the surface of the tongue with erosions at the angles of the mouth.

**Figure 5:**
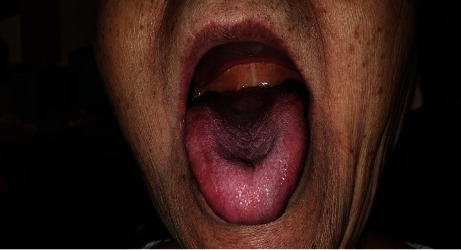
Appearance of oral cavity one month after discontinuing alendronate.

In view of the accelerated spread of the oral ulceration, the family decided to consult a dentist who referred the patient urgently to an oral and maxillofacial surgeon on the 22^nd^ of September (Figure 3). The surgeon diagnosed her to have an “allergic” reaction secondary to alendronate and asked her to no longer take alendronate. She was prescribed oral prednisolone, antiseptic mouth rinse, and antiseptic gel with some improvement of the oral ulceration and pain. She attended the metabolic bone disorders clinic on the 25^th^ of September. On examination in our clinic, she was found to be a well-nourished elderly lady with no evidence of systemic malaise. She complained of some pain on opening her mouth. Examination of the mouth revealed a well delineated 3 × 4 cm sloughy erosion with irregular margin affecting the hard palate and surface of the tongue with erosions at the angles of the mouth (Figure 4). On palpation, induration was felt along the margins. On further questioning, the patient's daughter stated that what she had understood of the instructions given to her by the pharmacist who dispensed the medicine was specifically that the medicine should be put in the mouth to be dissolved by saliva, to sit upright for 45 minutes and *then* to drink water. It was not clear to us if the instructions given had been misunderstood or if incorrect instructions had been given. Given the temporal association of exposure and our knowledge of potential chemically induced mucosal erosions with bisphosphonates, we made a diagnosis of alendronate-induced mucosal ulceration most likely caused by direct prolonged contact of the oral mucosa with the chemical agent. She reported to us 2 weeks later that she had fully recovered. A photograph taken at 1-month postepisode showed normal, edentulous oral cavity (Figure 5). This further confirmed our diagnosis.

## 3. Discussion

Oral bisphosphonates have been reported to cause oesophageal and gastric ulceration. However, bisphosphonate-induced oral mucositis and mucosal ulceration are uncommon, and only a few cases have been reported in the literature. These most often have been due to inability to take the medication correctly due to associated mental and or physical comorbidities [4–6]. The proposed mechanisms of mucositis and mucosal ulceration with bisphosphonates include the cytotoxic effect of nitrogen-containing bisphosphonates on the epithelial cells [7] as well as the direct irritant effect of the drug on the mucosa [6]. The lesions need to be differentiated from other causes of erosive ulceration including apthous ulcers and that due to trauma and infection. The most common cause of traumatic ulceration is due to ill-fitting dentures and sharp-edged crowns. Such ulcers usually do not demonstrate induration [8]. Our patient was edentulous and had her dentures for a long time prior and had not reported any problems with them before. Ulcers due to infections, for example, viral infections, generally occur as multiple small ulcerations. They present initially as fluid-filled vesicles, which then break down to form small ulcers with irregular margins. These may subsequently coalesce to form large irregular ulcers [8]. Apthous ulcers are usually small and round lesions covered often with a pseudomembrane and surrounded by erythematous haloes. The cause remains unknown though viral infections, B12 deficiency, etc., have been postulated [8]. Oral lichen planus usually presents as bilateral lesions on the buccal mucosa with a symmetrical lace-like pattern. Ulceration rarely may be seen within the lesion [8]. Pemphigus vulgaris, an autoimmune bullous disease, is characterized by bullae formation that subsequently develops into painful, shallow, irregular ulcers. Nikolsky sign—the pathognomonic sign of pemphigus—is usually present [9]. Our patient did not have any evidence to suggest that this was pemphigus vulgaris. She was also not on any other medications that cause oral ulcerations such as nonsteroidal anti-inflammatory drugs (NSAIDS), beta blockers, immunosuppressants, anti-inflammatory, or immunomodulating agents such as methotrexate [8].

Withdrawal of medication and symptomatic management are the mainstay of treatment for bisphosphonate-associated oral mucositis. It is imperative that healthcare providers provide detailed and proper counselling on the proper administration technique of oral bisphosphonates. Alendronate and other similar oral bisphosphonates should be taken first thing in the morning on an empty stomach *with a full glass of plain water* at least 30 mins to 1 hour before other food, drinks, and medications. Alendronate *should be swallowed whole* without chewing, and patients should remain in the upright position for at least 30 mins. Healthcare providers should avoid usage of alendronate in patients who cannot sit upright for 30 mins and in those with a history of dysphagia, reflux, and esophagitis. Reintroduction of alendronate is possible after the resolution of the ulcer and strict adherence to proper administration procedure.

## 4. Conclusion

Although oral bisphosphonates are generally well tolerated, oral mucosal ulcerations and mucositis can develop due to improper administration such as sucking, chewing, or holding it in the oral cavity for prolonged periods of time. Proper counselling and rigorous adherence to the administration instructions are key to prevent this complication. Osteoporosis is more common in elderly individuals, and they are more likely to be started on these medications. They often may not understand instructions, and especially in such patients, instructions should be reinforced, and patients should be encouraged to recite back to the physician or the dispensing pharmacist the instructions to make sure that they have understood them. No detail is too small when counselling patients on the proper administration of these potent medications, and it is the responsibility of the healthcare provider to ensure that the patient has understood the instructions. It is also important for healthcare providers to recognise this side effect to allow for prompt withdrawal of the medication upon its identification.

## Data Availability

No data were used to support this study.
